# Using interview excerpts to facilitate focus group discussion

**DOI:** 10.1177/14687941241234283

**Published:** 2024-02-28

**Authors:** Alina Geampana, Manuela Perrotta

**Affiliations:** 3057 Durham University, UK; 4617 Queen Mary University of London, UK

**Keywords:** Creative methods, elicitation, focus groups, interviews, discussion stimuli

## Abstract

The use of interviews and focus groups is well-established in the social science methods literature. However, discussion on how research can combine these two methods in creative ways is less common. While researchers are generally aware of the potential of focus groups for further probing issues that emerge in one-on-one interviews, few studies detail how this might be achieved in practice. In this article, we describe and reflect on a focus group elicitation strategy that uses individual interview excerpts to facilitate discussion in group settings. In our reflection, we draw on a study that investigated the sharing of embryo images in fertility treatment. The article contributes to the methods literature firstly, by reflecting on the novel use of individual interview material in focus groups and secondly, by discussing the re-enactment of interview excerpts as an effective audio elicitation tool to be used in the later stages of research.

The key aim of this article is to detail and reflect on an elicitation strategy whereby researchers use individual interview excerpts to facilitate discussion on the same topic in focus group settings. To outline the context and benefits of this approach, we draw on a study that entailed participant discussion about embryo image-sharing practices in fertility treatment. Below, we review the relevant literature, outline our research, and offer our reflection on this method.

## The role of focus groups and discussion stimuli in qualitative research

Focus groups allow researchers to gain insights into the extent of agreement between participants (Barbour, 2018; Morgan and Krueger, 1993). They also create a situation where participants may be required to explain their views to each other (Morgan, 1996, 2018). Demant (2012) describes focus groups as ‘active social experiments’ whereby power relations become visible through participant interactions. The focus group method has been instrumental in understanding challenging areas of medical practice (Barbour, 2005, 2018; Lester et al., 2005). Within medical research, it is also a useful tool to reach decisions in the delivery of healthcare services (Tan et al., 2010). Regardless of the topic of investigation, qualitative researchers need to be considerate regarding the fit of this method within the larger study design (Ayrton, 2019; Barbour, 2005; Kitzinger, 1994; Morgan, 2018). In social science studies, focus groups are most often used in conjunction with other methods, with individual interviews being a popular choice (Barbour, 2018; Morgan, 1996; Nyumba et al., 2018). In this article, we specifically approach focus groups as a method for deepening understanding of interview data and here outline a novel elicitation strategy to facilitate this process.

Recently, there has been considerable interest in developing activities and other innovative strategies to guide data collection in focus groups (Colucci, 2007; Cooper and Yarbrough, 2010; Rothwell et al., 2016; Zupan and Babbage, 2017). Visual prompts (Cooper and Yarbrough, 2010) as well as exercise-type activities (Colucci, 2007) feature among these approaches. Rothwell et al. (2016) stress the importance of participant knowledge on the topic of discussion and argue for an approach where interviewees receive evidence-informed information on the issue at hand; this can take the form of a video that participants watch immediately prior to the conduct of focus groups. Zupan and Babbage (2017) suggest that narrative text as well as film clips can be used to elicit emotion in focus groups. We here reflect on a research strategy whereby focus group prompts are used to further generate insightful discussion following individual interviews. We propose that re-enacted interview excerpts can be used effectively as a focus group elicitation tool. Such prompts consist of recordings of actors reading extracts from interviews conducted in the first research phase. The extracts then serve as elicitation tools in the second stage of focus group research.

Although the excerpts used in focus groups are not a vignette per se, they serve a similar function in that they allow for the discussion of an opinion/situation without participants feeling obligated to talk about their own struggles – this is especially important in research on emotional/complex topics (Aujla, 2020). Vignette-based approaches have been useful for both health research and research on sensitive topics (Sampson and Johannessen, 2020; Sheringham et al., 2021; Törrönen, 2018; Tremblay et al., 2022). Tremblay et al. (2022: p1) refer to vignettes as ‘short hypothetical accounts reflecting real-world situations’ and note that they can be used in both individual and group settings. Vignettes have been particularly useful in research to probe values and beliefs on a particular topic that is complex (Sampson and Johannessen, 2020). The focus on discussing a hypothetical and complex situation with re-enacted interview excerpts is similar to the motivations behind vignette research on sensitive topics (Aujla, 2020). However, while vignettes construct a situation, re-enacted interview excerpts provide an avenue to elucidate surprising interview findings without the need for researchers to create an appropriate scenario. Re-enacted interview excerpts also have more potential in conveying emotion, thus being particularly useful in discussing feelings and reactions. In our case, the focus of re-enacted excerpts is specifically to highlight the emotions expressed by participants themselves rather than the situational context of such views/feelings. While the use of focus groups as a follow-up to interviews is not new (Nyumba et al. 2018), we here suggest that interview excerpts can be used productively in group discussions to elicit deeper meanings attributed to emotionally complex topics – in our case, the impact of embryo images in fertility treatment.

## Research context and methods

### Embryos, images, and infertility treatment

During IVF treatment, patient embryos develop in the lab in incubators usually for five days before one or two are implanted in the uterus. Our study looked at the broad impact of new technologies that introduce cameras inside lab incubators, thus producing images and videos of the developing embryos. Such images and videos can be stored on computers and circulated between professionals in the clinic or to patients at various points in their treatment. Due to the novel nature of such technologies and the implications of potential emotional attachment to one's own embryos, stakeholders have not yet agreed on when is the best time to share embryo images/videos with patients, if at all.

Embryos can have multiple meanings depending on the context and group involved in creating that meaning. For example, our previous research (Perrotta and Geampana, 2020; Geampana and Perrotta, 2023) suggests that visual representations of embryos can be assessed in multiple ways. Professionals primarily aim to decipher implantation potential from such images/videos, while, for patients, visual representations might foster emotional connections to their embryos in addition to potentially indicating that a successful pregnancy will occur (Hamper and Perrotta, 2023). Research has shown that experiencing infertility affects one's sense of self and identity regardless of gender (Arya and Dibb, 2016; Greil et al., 2010). Although women's perspectives have been more extensively studied, research from the last decade is increasingly investigating men's infertility struggles as well (Ying et al., 2015). Thus, the context in which embryo images/videos are engaged is one of intense pressure and hope for a successful treatment. Embryo image-sharing practices vary considerably among clinics (Geampana and Perrotta, 2023). While some patients might see a picture of their implanted embryo at the time of transfer, others might see images/videos of their embryos following implantation, when a pregnancy is confirmed, after a live birth, or not at all. This is because embryo imaging technologies are relatively new and expensive, while U.K. fertility practice is highly heterogeneous, meaning that clinics use such technologies in different, not yet standardised ways. This heterogeneity motivated us to avoid a vignette depicting a ‘typical’ situation and rather present the variety of feelings/views provided by our interview excerpts.

### Methodology

Following the completion of 94 one-on-one interviews (51 with patients and 43 with professionals), the research team met to discuss how to explore shared meanings of embryo images in focus groups. The interview transcripts contained rich data summarising the tensions around embryo imaging practices, specifically how/when they should be shared with patients and how they should be interpreted by professionals. While increased knowledge of embryos can be useful, such videos/images are also emotionally charged. It is these two sometimes conflicting aspects that we wanted to further explore in the focus groups. We considered focus groups as a key method for looking at group dynamics (Ayrton, 2019) and how they affect views on research themes emergent from individual interviews (Kitzinger, 1994; Morgan, 1996, 2018).

The research team curated a list of interview excerpts that could be used to elicit thoughts and discussion in the focus groups. Once the elicitation excerpts were decided on and finalised by the team, we proceeded with hiring actors who could re-enact the excerpts. It was important to use actors in order to preserve the anonymity of participants. We also ensured that the excerpts used did not contain any potentially identifying information. For professionals, this meant ensuring there is no specific reference to the lab's procedures or location, while for patients, it meant not including any information relating to where they had their treatment or what their outcomes were. We organised a session in which the two actors read and rehearsed the excerpts. The research team provided some context for their interpretation and feedback on their re-enactment. A total of six re-enacted interview excerpts were produced and stored as audio-recorded files.

Researchers organised a total of six focus groups: three with patients and three with professionals. Four of the six group discussions were conducted on clinic premises in 2019, while the other two were conducted online in 2021. Each focus group had between five and seven participants. All professional focus groups plus one patient focus group were recruited through National Health Service (NHS) sites. Two further patient groups were recruited via an online survey distributed on relevant social media, in which participants were given the option to leave their contact details in case they were willing to be contacted for further research participation. The majority of patient participants were female. Only three were male and all attended the focus group with their female partner. Two couples attended the in-person patient focus group and one attended one of the two online patient focus groups. One online focus group did not have any couples participating. Excluding couples, focus group patients did not know each other prior to participating. Discussions indicated that patients were at various stages of their IVF treatment, ranging from beginning treatment to having already had a baby as a result of treatment. Professional focus group participants work in U.K. NHS clinics where our research took place. Each focus group included a mix of nurses, fertility consultants and embryologists.

There was no requirement that either professionals or patients need to have participated in individual interviews to be included in the focus group sample. Some of the focus group participants, most of them professionals, had also taken part in one-on-one interviews. This was due to the interviews and focus groups having been conducted in the same fertility clinics on-site. Because of the time constraints of the research project and NHS resources, it would have been unfeasible to limit patient focus group participation only to those who had already participated in individual interviews. Only one patient participated in both individual interviews and an online focus group. There is limited research on the advantages and disadvantages of having the same participants in interviews and focus groups; when exploring this, researchers tend to probe for confirmation of views across the two methods, with some evidence to suggest that focus groups might influence views espoused in interviews if conducted prior to these, but not vice-versa (Morgan, 1996, 2018). However, the purpose of the study was not to probe for differences/similarities between focus groups and individual interviews. The latter served rather to deepen interview themes.

For the in-person groups, we set up the room to be equipped with speakers so that our elicitation extracts could be heard clearly. The online focus groups followed the same interview guide. During group discussions, we played a minimum of four re-enacted interview excerpts and asked for immediate reactions to them. Focus group leaders then probed further based on initial insights. Although focus groups were not mixed, both patients and professionals were able to discuss interview excerpts generated by participants in the other group. This facilitated dialogue between lay and professional perspectives while also creating safe spaces where patients would not fear medical judgement. This was especially important as we had concerns about patient self-censoring in a mixed group. Although at the start of the project, mixed focus groups were considered a possibility, we gained a greater understanding of epistemic asymmetries between professionals and patients as the research progressed. Patient deference to their doctors is well documented in the medical sociology literature (Murgic et al., 2015). Based on our interview findings, we speculated that, in a mixed group, patients were likely to defer to professionals’ interpretation of the embryo images. This is a situation we wanted to avoid. By having separate focus groups with patients and professionals, judgements on any patient opinions were avoided.

## Using re-enacted interview excerpts in focus groups

### Choosing elicitation excerpts that encapsulate key interview findings

To explore group views on issues brought up in one-on-one interviews, we needed to probe views on interview themes. At the same time, we wanted to avoid summarising interview findings in a way that oversimplified fertility care, given the variation in care practices we have outlined. Within our research study, consideration had to be given to the fact that the experience of infertility is an emotionally charged subject. For example, it is not uncommon for infertility patients to experience difficulties talking about their struggles, especially if their treatment has not resulted in a pregnancy (Ying et al., 2015). With embryos being the topic of our discussion, this raised concerns about patients potentially being uncomfortable talking about those that resulted from their own treatment. This made the interview excerpt elicitation technique important to the research because it allowed patients to talk about embryo images in a hypothetical manner, expressing opinions about the views of others. Thus, our motivations were similar to researchers using a vignette technique to probe views on a sensitive topic (Sampson and Johannessen, 2020; Sheringham et al., 2021; Törrönen, 2018; Tremblay et al., 2022). The interview elicitation excerpt is thus similar to a vignette in that it might highlight a ‘real-world’ experience, but it is also akin to presenting a narrative (Sools, 2013) as told by the participant. Because our interview data revealed a multitude of views on embryo image sharing, sometimes even within the same interview, it would have been difficult to distil key themes in particular scenarios as vignettes do (Sampson and Johannessen, 2020). Thus, we concluded that several interview excerpts are better suited to capture the ambivalent feelings expressed by participants.

The elicitation excerpts we picked for focus group discussion are a concise encapsulation of tensions revealed in individual interviews in relation to embryo image sharing. Research team members first familiarised themselves with interview data. The use of grounded theory principles (Strauss and Corbin, 1997) during analysis facilitated early familiarity with codes and themes emerging from the research. When deciding which themes to elucidate further in focus groups, we focused on those where more data were needed and/or those that would benefit from the exploration of group dynamics. Analysis of interview data revealed that both professionals and patients experience worry about the sharing of embryo images. Professionals’ worries centred around (1) uninformed judgement of embryo implantation potential and (2) emotional attachment that can lead to a greater disappointment in the event of an unsuccessful IVF cycle. Patients reflected on the tension between wanting a visual representation of their embryos and the emotional attachment that comes with such artefacts. Picking appropriate excerpts was a team effort that consisted of narrowing down from a long list. Through continuous consultation, the team picked six excerpts – three from professionals and three from patients – to use during focus groups out of an initial list of 40. We aimed to choose excerpts that are vivid illustrations of the concerns expressed by participants.

To convey emotion in the elicitation excerpts, actor re-enactment was key. Actors were recruited through the researchers’ university's undergraduate drama programme and were compensated for their time. Researchers explained the study and its context, thus ensuring that the two actors adopt an appropriate tone. Re-enactment by voice actors fulfilled two functions. Firstly, although participants gave their consent for their data to be used for further research, it was still important to preserve participants’ anonymity. This meant that we needed to be careful that their voices were not recognised in focus groups. It would have been unethical to replay the original interviews; the voices had to be changed. When using such a technique, we suggest that researchers carefully consider whether participants can be identified from interview excerpts. Secondly, the actors were able to provide a clear and emotionally dramatic re-enactment. The recording entailed several takes to ensure clarity and impactful intonation. What resulted was audio material that conveyed emotion and drew listeners in, while also staying as true as possible to the original recording. Researchers may consider the possibility of using texts as stimuli (Stacey and Vincent, 2011; Törrönen, 2002), for example, a printed interview excerpt as opposed to audio material. Stimulus texts can be used in research to offer points of comparison for participants (Törrönen, 2002). However, when considering our excerpts, we noticed that intonation in the interviews entailed dramatic changes, with pauses, and variation in rhythm and tone. Due to this and the length of the excerpts, we decided a recorded version would be more impactful. The recording also has the advantage of all participants engaging with the stimulus at the same pace, while reading a text might take longer for some than others. Below are two examples of excerpts that were re-enacted and used as elicitation tools during focus groups.

**Professional elicitation excerpt**

Do you think that [sharing information/pictures of embryos] is a terrible idea?

Yeah, I think that's a bad idea. Having something which they can cling onto would be dangerous. Especially I have this one case in my mind of this person who had a 5AA back and she had a 4BB frozen and she wanted pictures of both of them. So I gave her the 5AA and I was like this is the one you’ve had back and she asked for the frozen one too. So she was just like that one is ugly compared to this one. And then she didn’t get pregnant with the first one, and now she's coming back for the frozen. The 5AA was just a textbook embryo, so it looked nice and round and lovely, and this one was just a big squidged. So yes, she is now thinking why am I going to get pregnant with this one after the last one looked so amazing. So it's a bit dangerous I think to show them. We had no qualms at all about putting it back, it was perfectly normal but it just wasn’t this textbook embryo. So yeah, it's a bit difficult to try to explain to patients that they can still get pregnant from this.

Have you had any other patients make comment about their embryos?

Not massively. Like I say a lot of people don’t really understand it when they look at it. They just see it as a ball of cells. We point out this forms the baby, this forms the placenta but they don’t know that cell structure like we do so it doesn’t mean a lot. You can obviously tell if we show them against the cartoon, this is what a perfect image is and this is what yours looks like. That's how she obviously knew hers was great but I think most people have no clue what it means.

Here the professional explains how sometimes neither the image of the embryo nor its scientific grade (‘5AA’ or ‘4BB’ in this case) reflects its implantation potential. They describe one of the embryos as ‘round and lovely’ while the other one is ‘squidged.’ The circulation of embryo images, especially ideal textbook types, reinforces normative judgements about embryo potential. However, many unknowns remain in the science of predicting pregnancy outcomes based on embryo features. Thus, the embryologist is concerned about patients judging embryos based on an image they received. The proliferation of embryo images and technologies that analyse them, to the respondent, presents challenges in cases where patients make rash judgements based on incomplete facts. In this excerpt, there is also the assumption that the embryo becomes an entity that patients ‘cling to’ or they become emotionally attached to very early in their treatment. Such concerns were expressed by many professionals in individual interviews. However, anecdotal evidence is too often presented as justification for professional fears around the sharing of embryo images. The excerpt then can lead focus group participants to reflect on key issues such as: (1) how, when and if embryo images should be shared; (2) the role of professionals in providing information to patients and (3) the relationship between image sharing and emotional attachment and anxieties.

**Patient elicitation excerpt**

Maybe not everybody is so affected but I feel a connection with those embryos. It's like oh God, those are ours, we’ve made those. Okay, the lab has done all the work but those are ours, you know. I felt really protective when they were transferred here. It was a real horrible day for me. I was thinking oh, my God, what if something happens to them. So actually to have been able to see, especially for the ones that made it to blastocyst, to have been able to see that would have been really nice. But then I don’t know if I’m saying that and then not being successful would have made it harder. Not quite sure how I’d feel about it if it didn’t work because then you have to disconnect yourself from what you’re seeing and, you know, I really find that hard to do just from seeing the little image of the embryo. It's already hard to go oh, but I saw that and that was ours and now it's just gone. I think that's quite hard to think about. So the more we see and then it doesn’t work, maybe the harder that would be. But then I guess it would be nice to have a choice if we have a successful birth. Then it would be nice to say do you want to go back and see the embryo start? That would be, that would be amazing for me. But I guess it will be interesting to see the journey of that embryo but yeah, if you kind of see that embryo going into six-cell and then it doesn’t go any further that's really sad and you’re already sad. And you didn’t even see it. So I don’t know if I’m just being a bit emotional about things, emotionally connected to them.

Many patients provided a reflection on their emotional connection to embryos in one-on-one interviews. In one of the excerpts chosen (provided above), an interviewee expresses ambivalence between wanting to see the embryo (‘something that they’ve created’) and this leading to an attachment that would make the emotional outcomes of a failed IVF cycle that much harder. This encapsulates how patients oscillated in interviews between excitement about seeing embryos and worry about personifying ‘a ball of cells’ before this has resulted in a successful pregnancy and birth. On one hand, this echoes professional concerns, but on the other hand, it adds an element of excitement and novelty to engaging with embryo images. This excerpt in particular facilitated group discussions of complex emotions experienced by patients.

### The interview excerpt as a ‘compare-and-contrast’ tool

The use of elicitation excerpts prompted a diverse discussion of emotional experiences during infertility treatment. For example, the patient excerpt that we provided in the section above sparked a discussion amongst patient participants on whether it is the images that foster emotional connections to embryos/sperm/eggs or rather attachment is already present regardless of the visual aids participants had at their disposal:

P1: I don’t know, I could, I like to feel I’ve got the picture of it because I’ve got the picture of it and then that worked, yeah. I don’t know. I got very attached straightaway to the eggs.

P2: I agree. You’re already attached anyway pre-pictures, even at five days when it went from 10 to seven to one and back to three and yeah, they’re dots but you’re definitely feeling it then.

P1: And we got a letter through when, I think it was about six months to be fair about whether or not we wanted to keep our sperm on ice because we had to have it surgically removed for (child). It's his badge of honour, he's ever so proud of it. (…) I don’t want to be pregnant again but I still couldn’t quite bring myself to let them take them out of the freezer, so I wrote back and said why don’t you just keep them for another year. Because it took us thirteen years to come round to having a child. (…) I can’t bear to get rid of his sperm yet so if I can’t get rid of his sperm, formed an emotional attachment to them, let alone the eggs and the embryos.

P4: The thing is though they’re safe in the freezer, aren’t they? If we do want to do it again we’ve got sperm that we have not got to go through stabbing me in my testicles.

P1: I am attached to them. Why would you destroy the sperm?

P3: I don’t know with the picture so I got it afterwards and we put it just like with the baby photos when you’re doing all the ultrasounds later and also with this one. I always feel like I kind of throw them in my bottom of my sort of desk drawer because I feel like I don’t want to get attached and like I don’t want to care too much because it's all very abstract and when I just kind of like I chuck it in the drawer and it's like well, you know, maybe I’ll take it out later and make an album. So it's kind of the opposite. Yeah, it's like it's sort of, you know, keeping a distance. (Patient focus group #1)

The excerpt prompted a compare-and-contrast of experiences of emotional attachment. Through group discussion, we were better able to understand not only that experiences differ, but that attachment is not necessarily dependent on the image but could be purely related to one's own biological materials regardless of the presence of the image in treatment. In the last paragraph, patient P3 suggested that keeping the image at a distance might provide some emotional relief from the stress of undergoing treatment. The power of the elicitation excerpt lies in the fact that it does not provide a straightforward answer to the question of whether an image intensifies attachment; it does not provide a typical situation or summary as a vignette might do. The elicitation excerpt rather draws on conflicting feelings and experiences. As such, focus group reactions to it prompted an analogous exploration of different factors that contribute to how patients encounter embryo images. This is not to say, however, that participants aimed to emulate the elicitation excerpt. Rather, by reflecting on a narrative that contained a multitude of emotions, they were encouraged to reflect on their emotions in a way that does not call for a simple conceptualisation of attachment to embryos and their representations. The elicitation excerpt opened up the possibility for answers that might challenge expectations. It also helps researchers avoid simple and/or leading questions. Playing the excerpt and asking participants how this relates to their own experience, we found, opened up the conversation considerably, especially in comparison to probing emotions in a one-on-one interview (Pascoe Leahy, 2022). Interestingly, responses in the focus group extract above also clarified that singling out the image as a factor in emotional responses does not necessarily capture the heterogeneity of participant responses.

Thus, the interview excerpt offers participants an opportunity to ‘re-perform’ emotions (Spowart and Nairn, 2014). The exploration of emotions is a key component of many qualitative studies (Cottingham and Erickson, 2020; Kearney and Hyle, 2004; Roach Anleu et al., 2016), especially in discussions of infertility (Carroll, 2013). In comparison to individual audio and photo diaries – techniques which can also be used to draw out emotions around a particular topic (Cottingham and Erickson, 2020; Spowart and Nairn, 2014), the use of interview excerpts in focus groups provide a more structured and group-focused discussion about views/emotions. As such, a researcher should consider whether this is the most appropriate venue to explore emotional topics or whether individual reflection would prove more fruitful. It is worth reflecting in the first place whether it is ethical to bring up and have complex discussions on topics which might cause emotional distress to participants. Specifically, it is important that participants know in advance what they will be asked to do and/or what will be discussed. They, of course, should have the option to not answer particular questions. If emotional topics are approached in the research, the other ethical issue is whether to explore complex emotions in an individual or group setting. In making our decision, we factored in the broader context of infertility discussions which we detail in the next paragraph. We think that others might also want to consider the private and/or public nature of what is being discussed and to what extent participants might benefit from sharing thoughts in a group setting.

In considering whether to use the excerpt technique, we contended that infertility is already an experience that has benefitted from institutional group support and discussion. For example, in the United Kingdom, there are numerous organisations and patient groups with a focus on sharing the emotional hardships of undergoing infertility treatment. Our choice was also influenced by the limitations of one-on-one interviews in prompting reflection on emotional aspects around embryo images. We found that being presented with someone else's reflection on embryo images prompts a compare-and-contrast reaction from participants – a reaction that is more difficult to draw out in one-on-one interviews. On the other hand, focus group dynamics (Ayrton, 2019; Demant, 2012; Farnsworth and Boon, 2010) did affect how each participant was able to express themselves. We found that some participants were more vocal and took up more space in the discussion. In particular, the inclusion of couples in one of the focus groups showed us that two individuals who have undergone infertility treatment together might be prone to talking to each other and reinforcing their experiences throughout the discussion, thus potentially making other participants with different emotional connections (or lack thereof) to their embryos as an outlier. Therefore, we conclude that consideration also needs to be given to participant similarities where this technique might be used. Particular to infertility patient participants, their emotions might be influenced by whether or not treatment was ultimately successful and/or whether they are still undergoing treatment.

### Elicitation as a strategy for reflection across epistemic divides

For the reasons discussed above, the researchers decided not to conduct mixed professional/patient focus groups. Nonetheless, we reflected on the need for cross-communication to bridge the well-documented epistemic divide between patients and professionals (Blease et al., 2017; Naldemirci et al., 2021). The discussion of professional elicitation excerpts by patients and vice-versa proved to be a useful strategy for prompting each group to reflect more deeply on their epistemic positions. Analysis of individual interviews in our study revealed tensions and variations between how patients and professionals think about embryos. New imaging techniques are meant to improve embryologists’ assessment of which embryo(s) might result in a pregnancy. The majority of professionals, however, expressed fears in individual interviews about the over-sharing of embryo images/videos that might lead to patient attachment and subsequent disappointment. On their side, patients understood the perils and pitfalls of early attachment to embryos but also had a limited understanding of the professional assessment of embryos. Professionals feared that too much information might be overwhelming for patients. Considering such differences in healthcare provision, some suggest methods like narrative elicitation for facilitating different groups’ understanding of each other, especially in reducing the professional judgement of patients (Naldemirci et al., 2021). Re-enacted interview excerpts can also, we suggest, be beneficial in focus groups with professionals, as excerpts paint the patient in a multi-faceted way. To aid in bridging epistemic divides, we specifically suggest that picking excerpts that might challenge stereotypes might be useful.

The response to the elicitation excerpts enriched our understanding of the dilemmas that professionals are confronted with in their daily practice. While in individual interviews the sharing of information about embryos and embryo images was a theme that was interesting yet underdeveloped, focus group discussion allowed for more in-depth discussion. Learning about the mixed emotions that patients go through during treatment prompted professionals to reflect on their own assumptions about patients’ needs, thus learning in the process and re-evaluating their previous assumptions which might have been epistemically problematic (Blease et al., 2017; Naldemirci et al., 2021). For example, in the focus group transcript below, a group of professionals reflected on how to respond to different patient needs with regard to providing information on embryos before and after implantation:

H4: Well, I find that when I listen to phone calls and things of the embryologist here they do tend to say like what stage they’re [the embryos] at on each day and go through it a little bit and open up an opportunity for like a bit of discussion or questions if that's what they want. So it depends where they are and what, who's speaking to them.

H1: I tend to divide them in to good quality, not so good quality and average, and then if they need more information sort of build on it from there.

Unknown: Yeah.

H1: Like what makes it a top quality or not so good.

H2: And then we talk about cell numbers as well. So like on day three we expect each embryo to have a minimum of five cells, for example. And then we’ll say we looked at all your embryos and three out of five of them have got eight cells, one has got seven cells, the other one has got six cells. So we do that as well.

H3: So I am actually wondering, right, after this discussion whether we should ask patients how much information they want. Because some patients might not want hardly any information but we give it to them and then it could stress them out, you know, if they think oh, what does this mean or last time I had this and now they’re not and they might get anxious.

H4: I guess that's the benefit of like doing what you said where you start off just describing what the quality is.

H3: Yes, it might be nice to have a discussion before like the egg collection and say how much information would you like? Do you want us to give you the bare minimum or would you like us to go into detail? Because also some of them might not be confident enough to say actually, please don’t tell me the grades. (Professional focus group #1)

While in individual interviews, this group assumed that patients might be overwhelmed by too much information, upon listening to the re-enacted elicitation excerpts they reflect on tailoring treatment, thus showing that they have slightly changed their position because of the prompts used in this research. The tone of the focus group discussion was more mindful of patient perspectives when compared to some assumptions they had made in individual interviews because professionals were able to share different clinical experiences and listened to elicitation excerpts from both patients and peers. We contend that the approach taken had two main benefits: (1) probing for views on a particular issue that had arisen in interviews and (2) bringing the patient’s voice into professional discussions. The latter has allowed for a deeper reflection on care practices than individual interviews had allowed.

As Linden and Singleton (2021) note, it is important to pay attention to ‘neglected things’ in care practices through research – in this case, the variety in patient views with regard to their treatment. Through the elicitation technique used here, we were able to give patient needs and concerns a more tangible form in discussions with professionals. We would stress that, to do this, it is necessary to highlight ambivalence as patients will have different experiences. We wished to avoid suggestions of patient uniformity of views in the elicitation excerpts hence why these showed a variety of emotions related to images and videos of embryos. Similarly, Blease et al. (2017) suggest that it is important that research highlights that the experiences of both patients and professionals are quite mixed. This relates again to our wish to use re-enacted excerpts as opposed to vignettes (Aujla, 2020; Sampson and Johannessen, 2020; Sheringham et al., 2021; Törrönen, 2018; Tremblay et al., 2022) to avoid the suggestion of a ‘typical’ situation. Qualitative research in health can take many forms, but it is important that the researchers reflect on their practice in light of their questions and aims (Katz and Mishler, 2003). Elicitation excerpts from a lay or professional group, when presented to the other group, have the potential to bridge epistemic divides by allowing each group to reflect on another's thoughts, feelings and practices. Professionals might have certain styles of reasoning and articulation that make patient testimony seen as emotional and less credible (Carel and Kidd, 2014). We found that the articulation of thoughts expressed in the patient elicitation excerpts made professionals pause and reflect on whether infertility patients are a monolith category. We speculate that the re-enacted excerpt technique might have a wider application in cases where power differentials exist, thus adding to research that has emphasised the use of narrative elicitation (Naldemirci et al., 2021). Whether or not mixed groups are appropriate would depend on the particular context of the research being undertaken. In our study, focus group reflection on interview excerpts allowed for a safe space to voice concerns and uncertainty – something that is important if one is to explore how care in particular settings can be improved.

## Discussion/conclusion

In this article, we propose creative ways in which interviews and focus groups can be used together, particularly through the creative use of data generated. As others (Kitzinger, 1994; Morgan, 1996, 2018; Rosell et al., 2014) have noted before, interviews lack a venue for the emergence of common understandings of meanings in social processes. These shortcomings can be addressed through the use of focus groups. We have here shown how focus groups can build on interview themes through the discussion of re-enacted elicitation excerpts extracted from interviews. Firstly, this technique allows for further development of interview themes or novel findings that require a deeper understanding. Secondly, the technique allows researchers to reflect on initial interview findings during the research process and collect more data specifically on themes of interest. Thus, the interview elicitation technique is compatible with iterative research processes (e.g. grounded theory) whereby analysis takes place alongside data collection. It is not compatible with research where interviews and focus groups need to take place at the same time. Additionally, as the focus group questions build on interview data rather than asking participants the exact same questions that were asked in interviews, the technique does not allow for a direct comparison of results between interview and focus group data as others have done (Guest et al., 2017; Kruger et al., 2019). Rather, data collection in focus groups was meant to complement interview findings and further develop insights on topics of interest.

Although our research funding has allowed the hire of professional actors to re-enact the interview excerpts, we suggest that it would also be possible for researchers to re-enact and record themselves instead. Of course, a visual illustration of interview quotes would also be a possibility. However, as others have stressed (Caro, 2016; Zupan and Babbage, 2017), we also suggest that audio excerpts might be more effective in conveying emotions – this is particularly important if the topic at hand is conducive to emotive reactions and reflections. In this research, we think that a summary or a text reading of embryo image-sharing issues would not have had the elicitation impact the re-enacted excerpts provide. Previous research (Caro, 2016; Zupan and Babbage, 2017) suggests that audio and visual stimuli elicit stronger emotional reactions from participants than a text reading. Thus, audio prompts have greater potential to engage interviewees in discussions on emotive subjects (Zupan and Babbage, 2017). In practice, this technique also facilitated more open-ended reflections on connections between different themes as opposed to researchers dealing with each one separately.

One of the main advantages, in our view, of using the interview excerpt elicitation technique is the ability of excerpts to convey emotion in all its nuances as expressed by participants. Although we did consider using a more conventional vignette technique (Jenkins et al., 2010; Sampson and Johannessen, 2020), whereby researchers depict a relevant situation/scenario to be discussed with participants, we contented that it would be difficult to capture the multitude of emotions and connected issues that the sharing of embryo images gives rise to. As vignettes tend to present a typical or idealised type of situation (Sampson and Johannessen, 2020; Sheringham et al., 2021; Törrönen, 2018; Tremblay et al., 2022), the case at hand would not be suited for this approach due to patient care trajectories being very different from each other. We also wanted to avoid a discussion that was too abstract, not related enough to patient experiences and that would lead to views on social norms rather than concrete experiences (Sampson and Johannessen, 2020). When compared to vignettes, re-enacted excerpts allow more focus on emotions rather than the context of the patient's situation, which, in our research, differed quite widely.

We suggest that there is also a possibility of using interview elicitation excerpts in individual interviews or follow-up interviews. In this case, researchers should consider what topic they wish to explore further in interviews and whether the research design allows for the exploration of this topic in more than one stage – with the first stage being the one where excerpts for later use are produced. Researchers can also consider whether they would like to use the excerpts as a follow-up discussion with the same participant or use these as prompts with a variety of participants and not necessarily the ones that the excerpts originated from. Each of these options, we think, would open up a multitude of discussion possibilities which can be tailored to the needs of the specific research project.

Our elicitation technique also allowed for inter and intra-group reflection. This is especially important in order to create a safe space across epistemic divides, particularly in light of research (Blease et al., 2017; Carel and Kidd, 2014; Murgic et al., 2015; Naldemirci et al., 2021) showing that epistemic asymmetry is a poignant social issue, especially in fields like medicine. Following focus groups, professionals expressed to researchers that they appreciated the space and encouragement to reflect on what patients might want. We also conclude that patients were able to be more open about the care they received in the reflection space provided by patient-only groups. We suggest that elicitation excerpts combined with moderator skills can ‘translate’ difficult emotions/conversations to groups that see a certain issue from different points of view due to different modes of socialisation and perceived degrees of expertise. Although we do not know what the outcome of a mixed professional/patient group would have been, we structured our excerpt in a way that allowed for a conversation across groups. However, it is important that the conversation does not stop there. In the context of our project, we devised further public engagement events and outputs where dialogues between professionals and patients continued in more direct ways.

While focus groups still tend to be underused in social science research (Cyr, 2016), we contend that they have great potential to complement other qualitative methods. Existing literature (Ayrton, 2019; Barbour, 2005, 2018; Demant, 2012; Freeman, 2006; Kitzinger, 1994; Morgan, 1996, 2018; Rosell et al., 2014) emphasises how using focus groups allows researchers to explore group dynamics in addition to the patterns observed in the content of the conversation. Recently, we have seen more emphasis on devising engaging activities (Colucci, 2007; Cooper and Yarbrough, 2010; Rothwell et al. 2016) for focus groups. We here suggest that re-enacted interview excerpts can also be used as a means to engage participants in lively discussion. Although our approach does not constitute a practice-oriented activity per se, it does give participants a concrete narrative to discuss. This has the advantage of allowing participants to discuss their own experience/emotions if they wish to do so but does not make it a requirement in the conversation. Although our proposed research approach is not applicable to all contexts (e.g. where interviews and focus groups run in parallel with each other or where focus groups are conducted first), it furthers methods and strategies for integrating interviews and focus groups as well as techniques for effectively engaging focus group participants in the discussion of emotionally sensitive and/or complex topics.
